# Causes for Rejection of Keratorefractive Surgery in a Central Indian Population

**DOI:** 10.7759/cureus.16179

**Published:** 2021-07-04

**Authors:** Rajesh S Joshi, Ashok H Madan, Tanmay Surwade, Pranshu Goel

**Affiliations:** 1 Ophthalmology, Government Medical College and Hospital, Nagpur, IND

**Keywords:** keratorefractive surgery, laser-assisted in situ keratomileusis, photorefractive keratectomy, rejection for refractive surgery, refractive surgery

## Abstract

Aim

To identify the reasons for refusing refractive surgery in patients visiting for spectacle-free vision.

Methodology

Medical records of 296 patients who presented for keratorefractive surgery (KRS) from June 2017 to April 2020 at a tertiary eye care center in central India (Government Medical College and Hospital, Nagpur, Maharashtra, India) were reviewed. Demographic details of all the patients and parameters obtained during workup of a case presented for KRSs were captured in an Excel^®^ sheet and analyzed statistically.

Results

Of the 296 patients who presented for KRS during the study period, 86 (29.1%) patients were denied KRS. The mean pachymetry in the right eye was 505 μm ± 10 μm (range 520-485 μm) and 502 μm ± 7 μm (511-490 μm) in the left eye. Suboptimal corneal thickness (n = 28, 32.6%) was the most common reason for rejection. Other reasons for not recommending the procedure were high myopia (n = 20, 23.3%), spectacle not stable (n = 16, 18.6%), and keratoconus (n = 11, 12.8%). Collagen vascular diseases (n = 3, 3.5%) and anxiety about the procedure (n = 2, 2.3%) were causes unrelated to the procedure. No correlation was observed between corneal thickness and degree of myopia (r = 0.014, p = 0.66).

Conclusion

Patients presenting for KRS exhibit various problems. Meticulous preoperative evaluation is most important for long-term visual outcome. Suboptimal corneal thickness, high myopia, unstable spectacle correction, and keratoconus were the common reasons for not performing KRS in the study population.

## Introduction

The development of excimer laser has improved the safety and efficacy of keratorefractive surgery (KRS). However, the long-term success of laser-assisted in situ keratomileusis (LASIK) and photorefractive keratectomy (PRK) depends on appropriate preoperative assessments. LASIK and PRK are cornea-based procedures. Therefore, the decision of performing KRS in patients is based on adherence to the set parameters to assess the cornea. All patients aspiring for spectacle-free vision may not benefit from the procedure, and patients with high myopia and thin corneas are not suitable for refractive surgery. Therefore, alternative procedures for spectacle-free vision such as implantable contact lenses or lens-based surgery may be advisable for such patients.

In the present study, we identified the reasons for not performing refractive surgery in the central Indian population and determined whether any correlation exists between the corneal thickness and degree of myopia. Although we identified a few reports that have elaborated on the reasons for not performing refractive surgery in candidates presenting for surgery [1-6]. No study has been conducted on the central Indian population so far.

## Materials and methods

This retrospective, observational, record review study was conducted in a tertiary eye care center in central India after obtaining approval from the institutional review board. The study was conducted in accordance with the tenets of the Declaration of Helsinki. Medical records of the patients who presented for refractive surgery between June 2017 and January 2021 in a refractive surgical unit situated in central India were retrieved. Details of age, gender, visual acuity, uncorrected and corrected visual acuity, refractive error, slit lamp biomicroscopy, dilated fundus examination by indirect ophthalmoscopy, intraocular pressure, corneal topography (Pentacam; Oculus, Arlington, USA) and type of refractive surgical plan (LASIK or PRK) were recorded. If the central corneal thickness was >480 μm and residual stromal bed was >290 μm, KRS was advised. Myopia more than -8 D, hypermetropia and astigmatism more than 4.0D were not considered for the KRS. Table 1 summarizes the screening criteria followed in our refractive surgery center. Refractive surgery was not performed in the patients who did not meet these criteria. In case the surgery was not performed, the reason was noted. All cases were performed using wavefront-guided VISX Star S4 Custom Vue (Johnson and Johnson Vision, Santa Ana, CA). Eye tracker evaluation and iris registration were performed for both the eyes of each enrolled patient.

**Table 1:** Criteria for selection of candidates for refractive surgery. KCI: keratoconus index.

Criteria for refractive surgery	Characterization
Age	>18 years
Myopia	-8.0 D
Hypermetropia	<4.0 D
Astigmatism	<4.0 D
Central corneal thickness	>480 μm
Residual stromal bed thickness	>290 μm
Stable refraction	For one year
Keratoconus	KCI > 5%

Statistical analysis

The data were entered in an Excel® sheet (Microsoft Corporation, Redmond, WA, USA), and statistical analysis was performed using SPSS version 13.0 (SPSS Inc., Chicago, IL, USA). Continuous variables are expressed as mean ± standard deviation and categorical variables are expressed as percentages. Spearman’s correlation test was used to determine the correlation between central corneal thickness and degree of myopia. 

## Results

A total of 296 patients presented for KRS during the study period, and 210 patients(119 females and 91 males [F:M, 1.3:1]) were considered for KRS (70.9%). Of these 210 patients, 200 patients underwent LASIK (95.2%) and 10 patients were considered for PRK (4.8%). KRS was not performed in 86 (29.1%) patients. The mean pachymetry value in the right eye was 505 μm ± 10 μm (range, 520-485 μm) and 502 μm ± 7 μm (range, 511-490 μm) in the left eye. The reasons for rejection are presented in Table 2. Suboptimal corneal thickness (n = 28, 32.6%) was the most common reason for rejection. Other reasons for not recommending the procedure were high myopia (n = 20, 23.3%), spectacle not stable (n = 16, 18.6%), and keratoconus (n = 11, 12.8%). Surgery was not performed in a few patients because of multiple reasons: two patients had high myopia, low central corneal thickness, and keratoconus; two patients had high myopia, low central corneal thickness, and myopic maculopathy; and one patient had high astigmatism, thin cornea and keratoconus. No correlation was observed between the central corneal thickness and degree of myopia (r = 0.014, p = 0.66).

**Table 2:** Reasons for rejection of patient for keratorefractive surgery.

Reasons for rejection	Number of patients (%)
Suboptimal central corneal thickness	28 (32.6)
High myopia (>11.0 Diopters)	20 (23.3)
Spectacle not stable	16 (18.6)
Keratoconus	11 (12.8)
Herpetic keratitis history	6 (7)
Collagen vascular diseases	3 (3.5)
Anxiety about procedure	2 (2.3)
Total	86

## Discussion

LASIK and PRK are two commonly performed procedures for correcting refractive error. The safety and efficacy of both the procedures have been established in various studies [7,8]. However, the success of both these procedures depends on accurate preoperative assessments. KRS is usually performed on young eyes. The expectations from the procedure are high. Large variations have been observed in corneal parameters across the world [1,3-5]. In the present study, we aimed to explore the reasons for not performing KRS and the correlation between the degree of myopia and corneal thickness. 

Although LASIK and PRK are safe procedures for ensuring spectacle-free vision, they are not ideal for all patients. For long-term safety and effectivity of these procedures, identification of factors that can harm vision in the long term is essential. In the present study, out of 296 patients, 86 patients (29.1%) were rejected because of various reasons. Previous studies have reported that the rejection rate varies from 21% to 34%. [1-6]. The most common reason for rejection in our study was suboptimal corneal thickness (n = 28, 32.6%). LASIK and PRK involve ablation of the normal corneal stromal tissue, leading to the weakening of the cornea. High myopia and thin cornea excessive ablation of the stroma lead to thin cornea. A thin cornea is always at a risk of developing keratectasia, which is irreversible [9]. In a study conducted by Brenner et al., 75.3% of the patients presenting with post-LASIK ectasia and demonstrated signs of forme fruste keratoconus, which certainly culminate in the development of ectasia [10]. The incidence of iatrogenic keratectasia has been reported to be more after LASIK than after PRK [11]. A few studies have shown that LASIK performed in a thin cornea with a pachymetry value of <500 μm is safe [12-14]. However, all corneas with the same pachymetry values do not necessarily have the same strength [15]. Spaeda et al. reported that the residual bed thickness (RSB) is an important factor for the development of ectasia after KRS [16]. An RSB thickness of <250 μm has been associated with the development of keratectasia [12]. Randleman et al. suggested that the risk of keratectasia development is present even when the RSB thickness is >300 μm. [11]. Padmanabhan et al. reported keratectasia in a patient with an RSB thickness of 327 μm [17]. In our study, we considered an RSB thickness of >290 μm. Sharma et al. also noted suboptimal corneal thickness (55.1%) as the reason for not performing KRS [3]. The RSB thickness in their study was ≥300 μm. A similar observation was made by Xu et al. wherein 28.6% of patients had low corneal thickness [5]. Hashmani et al. observed that an increased risk of corneal ectasia is the most common reason for not performing this surgery in Pakistan; [6] the authors reported that overablation of the cornea may lead to iatrogenic keratectasia, which could be difficult to handle because of the availability of limited resources for the treatment of this condition. 

High myopia (> −8.0 D) was found to be the second most important reason for rejection in our study. Yamane et al. reported that with an increase in the extent of myopia correction, the higher-order aberrations (HOAs) increase significantly, which compromises contrast sensitivity [18]. The increase in HOAs is associated with a decrease in day and night visual clarity, which may lead to diplopia, ghosting, and glare in these patients [19]. Correction of high myopia by LASIK renders the cornea weak for life. Therefore, in these patients, alternative treatments in the form of implantable contact lenses, which are safer than LASIK, are recommended. Bamashmus et al. [1] and Hori-Komai et al. [2] reported high myopia (>11.0 D) as the most common reason for rejection in their study. In our study, the upper limit of myopia correction was −8.0 D. On the other hand, some authors have kept a cut-off of up to −12.0 D [2,3]. Consensus regarding the treatment of upper limit of high myopia is lacking. The thickness of the central cornea is also important. However, the optimal corneal thickness critical for predicting the development of ectasia has not been determined yet. We avoided any LASIK procedure in patients whose corneal thickness was <480 μm even if all the topographical parameters were normal. However, in a few studies, the corneal thickness of >500 μm was considered as the selection criterion for performing KRS [3,5]. Five patients had more than one unsuitable condition because of which KRS was not performed. These five patients had low corneal thickness commonly associated with high myopia, keratoconus, or high astigmatism. Sharma et al. in their study of 338 patients had 21 patients with more than one contraindication in various combinations [3].

The safety of LASIK and PRK has been reported to be up to 4.0 D of hypermetropia in a few studies [20,21]. As far as hypermetropia was concerned, patients with hypermetropia of more than 4.0 D were rejected because of the possibility of excessive steepening of the central part of the cornea and the likelihood of undercorrection. Alio et al. reported the risk of HOA development and the high enhancement rate (29.4%) in patients with hypermetropia of >5.0 D who underwent LASIK [21]. Bamashmus et al., Alsulami et al., and Hashmani et al., considered the upper limit for hypermetropia as 4.0 D [1,4,6], whereas Sharma et al. considered the limit as 5.0 D [3].

Unstable refraction (n = 16, 18.6%) was identified as the third most common reason for rejection in our study. In case of change in refraction on the first visit of 0.5 D either in sphere or in cylinder, we waited for one year for considering refractive surgery. Refractive surgery in such patients may lead to patient dissatisfaction in the future or may become the cause of an early sign of corneal ectasia. Alsulami et al. reported it as the most common reason for rejection in their study [4].

Keratoconus was found to be another common reason for rejection (n = 11, 12.8) in the present study. Unstable refraction has been reported after KRS even in patients with mild keratoconus [2]. Therefore, other alternatives for patients with mild or suspected keratoconus is advisable. Collagen cross-linking along with simultaneous surface ablation has been advised in patients with keratoconus [22]. However, information regarding the long-term safety of this procedure is not available in the literature. 

Patients with a history of herpetic keratitis (n = 6, 7%) were excluded from this study. The American Academy of Ophthalmology has listed herpetic keratitis as a contraindication for KRS. The incidence of herpetic keratitis is far greater in patients who have undergone PRK and LASIK than in the general population [23,24]. Hori-Komai et al. also excluded patients with a history of herpetic keratitis [2].

Non-ocular reasons observed in our study were collagen vascular diseases in three patients (3.5%) and anxiety about the procedure (n = 2, 2.3%). Schallhorn et al. in their retrospective study on KRS in patients with collagen vascular diseases reported that KRS can be performed in patients with well-controlled collagen vascular diseases [25]. However, its effect on the ocular surface during disease exacerbation is not known. Therefore, we excluded patients with a history of collagen vascular diseases.

Bamashmus et al. and Hashmani et al. have reported pregnancy as a reason for rejection for KRS [1,6]. Alsulami reported that out of 50 patients, 18 patients were rejected for non-medical reasons [4].

No surgery is perfect and can guarantee 100% results. Patients with unrealistic expectations and those who want perfect results should be avoided. We avoided such patients (n = 2, 2.3%) in this study. Therefore, non-ocular causes in addition to ocular causes of rejection should be considered.

Various authors have reported different reasons for rejection. Variations in the rejection rate and reasons for rejection could be due to differences in geographical locations and strategies exercised to collect the data.

We studied the correlation between the corneal thickness and degree of myopia and observed no correlation between these two parameters. The finding is similar to that reported in a study by Alsulami et al. [4].

The limitations of our study are its retrospective nature and selection of patients visiting to a single center for spectacle-free vision. We recommend conducting a prospective, multicenter study to collect more data related to rejection and to set uniform guidelines that will be useful for clinical practice. 

## Conclusions

In the present study, we report suboptimal corneal thickness as the common reason for rejection for KRS in patients. However, the same may not be true for patients from other geographical locations. Lastly, in addition to proper preoperative evaluation, patients’ psychological condition, which is important from the viewpoint of patient satisfaction after the procedure, should be considered. 
